# Supercritical Fluid Chromatography: Application to Trace Analysis

**DOI:** 10.6028/jres.093.096

**Published:** 1988-06-01

**Authors:** Milton L. Lee, Nebojsa Djordjevic, Karin E. Markides

**Affiliations:** Department of Chemistry, Brigham Young University, Provo, UT 84602

Supercritical fluid chromatography (SFC) is fast becoming the method of choice for many separations involving nonvolatile, reactive, and thermally labile compounds. In particular, supercritical carbon dioxide has found wide applicability as the mobile phase in SFC because of its low critical parameters, mild chemical properties, and excellent compatibility with a variety of detection systems. Supercritical fluids have lower densities and, hence, yield higher chromatographic efficiencies for equal analysis times when compared to liquids, and they possess solvating power to modify retention instead of relying on temperature for this purpose such as in gas chromatography.

Most SFC work in the past has been more qualitative in nature than quantitative. This is a result of the newness of the technique and efforts to explore the full range of its applicability. As the technique has become an established member of the analytical arsenal, more emphasis on quantitative aspects has arisen.

The most important contributing factors to the use of SFC in trace analysis are (a) sample introduction, (b) column inertness and resolving power, and (c) detector sensitivity. Packed columns have traditionally been preferred for trace analysis in chromatography because larger sample volumes could be injected onto such columns, thereby providing greater target analyte amounts for detection. However, these high surface area packings often exhibit some adsorption and/or catalytic decomposition of sensitive compounds, leading to lower sensitivities and reproducibilities. Furthermore, the carrier fluid flow rates are often too large to introduce directly into the desired detectors, and splitting of the column effluent is required. This again reduces the sensitivity, A compromise is achieved by using microbore columns packed with low surface area, highly deactivated microparticulate packings.

Capillary columns have the advantages that chromatographic peaks are narrower, the columns can be made more inert, and direct coupling, without splitting, to detectors is usually straightforward. Using the new dehydrocondensation deactivation reagents to mask surface silanol groups during column manufacture, polar compounds can be chromatographed using supercritical carbon dioxide with little or no reversible and irreversible adsorption at the subnanogram level. With such columns, underivatized carboxylic acids [2], steriods [3], polar pharmaceuticals [3], and drug metabolites have been successfully analyzed.

Polysiloxane stationary phases containing carefully designed organic pendant groups have broadened the scope of selectivity in capillary SFC. The incorporation of *n* -octyl, biphenyl, cyano, and liquid crystalline groups onto the polysiloxane backbone has increased the variety of selective stationary phases [4]. The biphenyl group has a very strong dipole induced-dipole character, while cyano groups are moderately polar as well as strongly polarizable. Geometric orientation is the interactive force that is characteristic of the liquid crystal groups. These useful interactive forces have demonstrated that the selection of stationary phases for use in SFC has a great influence on the separation of critical solutes.

The extremely low mobile phase flow rates (several microliters per minute) encountered in capillary SFC have encouraged the evaluation of a variety of detection systems for this technique. Particularly noteworthy is the relative ease experienced in adapting the flame ionization detector (FID), which is by far the most popular detector in gas chromatography, to capillary SFC. The main obstacle that had to be overcome was the decompression of the supercritical fluid at the column outlet through a carefully designed flow restrictor. The only apparent limitation is the required use of inorganic mobile phases that do not give responses in the detector.

Other flame-based detectors, thermionic [5] and flame photometric [6], which are also common to GC, can be used in capillary SFC. The only major limitation is the limited choice of mobile phases. Even CO_2_ gives a weak response at the sulfur wavelength in the flame photometric detector and reduces the detector sensitivity by one order of magnitude. With nitrogen-selective thermionic detection, picogram detection limits can be obtained [5].

The optical detectors common to LC, i.e., uv-absorbance [7] and fluorescence [8], do not suffer from the problems of flow restriction (the restrictor is placed after the detector) common to the flame-based detectors. However, the small detector cell volumes necessary for the small diameter (<100 µm) capillary columns lead to reduced sensitivities. Novel detector cell designs incorporating focusing optics and optical fibers have greatly eliminated these problems, and very sensitive detectors are available. Again, picogram detection limits can be obtained for favorable compounds using a fiber-optic based fluorescence detector [7].

The greatest challenge in coupling SFC to detection systems is the successful interfacing to information-rich spectroscopic instruments such as mass spectrometers and infrared spectrophotometers. In the case of mass spectrometry, decompression into a vacuum must occur before fragmentation. Recently, detection limits of low picograms in the full scan mode, and low femtograms in the selected-ion mode, were obtained using a coupled SFC-high resolution mass spectrometer system [9].

Sample introduction is the most difficult aspect of trace analysis in SFC. The sampling modes most widely used require splitting of a set volume delivered from a high pressure sample loop. This requires rather highly concentrated samples. Recently, solute focusing methods utilizing a retention gap, analogous to such methods used in capillary gas chromatography, have been applied in capillary SFC [10]. Proper manipulation of pressure and temperature is essential to achieving good chromatographic performance upon injection of large sample volumes. With 1-µL injection volumes, sub-ppm concentrations can be detected using an FID, and much greater detection limits can be achieved using more selective detectors.
